# The interrelationships between parental wisdom, psychological resilience, and their children’s emotional competence: a network analysis in the family context

**DOI:** 10.3389/fpsyg.2026.1709744

**Published:** 2026-05-07

**Authors:** Abdulaziz Mohammed Alismail, Lamia Abdullah Aladsani

**Affiliations:** Department of Education and Psychology, College of Education, King Faisal University, Al-Ahsa, Saudi Arabia

**Keywords:** network analysis, parental wisdom, psychological resilience, emotional competence, family systems

## Abstract

**Introduction:**

This cross-sectional study investigated the structural relationships among parental wisdom, psychological resilience, and parent-perceived children’s emotional competence using network analysis within Saudi Arabian families (*N* = 252 parents: 95 fathers, 157 mothers; ages 20–69 years).

**Methods:**

Participants were recruited through multi-channel sampling (digital platforms, community centers, snowball referrals) across multiple Saudi regions over 3 months. Parents represented diverse marital durations (49.2% married 11–20 years) and reported on 252 children (62.3% males; 42.5% aged 10–15 years). Validated Arabic measures included the 3D Wisdom Scale, Resilience Evaluation Scale, and Parental Assessment Questionnaire for Children’s Emotional Competence (McDonald’s ω = 0.728–0.924).

**Results:**

A GLASSO network estimation identified seven nodes across three communities (wisdom, resilience, emotional competence), exhibiting moderate edge density (0.667) and small-world properties (SWI = 1.028). Self-confidence and self-efficacy showed the highest strength centrality (1.008 and 0.952, respectively), suggesting their potential importance within the network structure. The self-confidence–self-efficacy connection demonstrated the strongest association (weight = 0.60), while affective wisdom appeared to function as a bridge linking parental resources to children’s emotional competence. Stability analyses indicated acceptable robustness for strength centrality but limited overall stability (CS-coefficient = 5.2%). Accordingly, findings related to betweenness centrality and bridge roles should be interpreted with caution and considered preliminary pending replication with larger samples.

**Discussion:**

Key limitations include the cross-sectional design, which prevents causal inference, reliance on parental proxy reports, which may introduce bias, and cultural specificity, which constrains generalizability. Nevertheless, the findings offer meaningful implications for Vision 2030–aligned family policy development, including: (a) prioritizing self-efficacy enhancement as a potentially high-leverage intervention target; (b) integrating resilience- and wisdom-based training to promote family cohesion; and (c) implementing culturally adapted parenting programs that emphasize affective wisdom and Islamic principles (ra _._hmah, tawakkul). Overall, the results contribute to the growing application of network psychometrics in Arab family contexts while highlighting the need for further validation and replication.

## Introduction

The family constitutes the foundational ecological system within which children’s physical and psychological development unfolds, exerting profound and enduring effects on long-term health trajectories and psychosocial wellbeing (Ren et al., 2022). Empirical evidence consistently demonstrates that supportive parenting practices, characterized by flexibility and responsiveness, serve as protective factors against personality disorder development, whereas suboptimal parent-child interaction quality precipitates elevated psychological and physical health risks (Ren et al., 2022). This bidirectional influence underscores the imperative of strengthening positive family bonds to ensure optimal developmental outcomes for children across their lives.

### Theoretical foundations: parental wisdom, psychological resilience, and children’s emotional development

In this study, wisdom comprises three core domains: the *cognitive domain* (rational analysis and sound judgment), the *reflective domain* (self-awareness and perspective-taking), and the *affective domain* (emotional understanding and empathy) (Sternberg and Glück, 2022). Almarshoud (2020) emphasized cognitive elements of wisdom, defining it as accurate self-perception, adaptive management of life situations, and rational decision-making. Al-Qurashi (2023) underscored the affective dimension by describing wisdom as higher-order mental abilities that integrate emotional and moral reasoning to promote collective welfare. Wasif (2022) advanced the reflective dimension by identifying wisdom as evolved thinking developed through self-knowledge cultivation and emotion-regulation mastery. Collectively, these perspectives establish wisdom as a sophisticated psychological resource grounded in intrapersonal and interpersonal understanding (Al-Hawari and Al-Khusussi, 2018).

Within family contexts, parental wisdom serves as a critical relational resource enabling parents to navigate complex family challenges through balanced reasoning, multiple-perspective consideration, and emotional attunement (Sternberg and Glück, 2022; Tarhan and Ünal, 2024). Wise parental decision-making reduces intrafamilial conflict and enhances mutual understanding (Dumbravă, 2017). Parents who exhibit higher levels of wisdom demonstrate superior emotion-recognition skills and provide more appropriate emotional support, contributing to increased family harmony (Xiao, 2025). Zare Mazloom et al. (2024) further demonstrated that parental wisdom significantly enhances children’s social intelligence and emotional competence, indicating potential intergenerational transmission pathways.

Psychological resilience—the second parental resource examined in this study—is conceptualized through two central domains: self-confidence and self-efficacy. Self-confidence reflects parents’ belief in their ability to withstand challenges, whereas self-efficacy involves mobilizing cognitive and emotional resources to manage stressful situations effectively. The integrative conceptualization adopted here synthesizes endurance, recovery, and post-adversity growth (Meseguer-de-Pedro et al., 2019). Alismail and Almulla (2023) describe resilience as a dynamic, process-oriented phenomenon characterized by perseverance and positive adaptation—properties that align closely with the self-confidence and self-efficacy dimensions. Contemporary stress-adaptation theories similarly contend that effective stress responses help maintain psychological and interpersonal stability within family systems (Kashdan and Rottenberg, 2010). Hendawy (2023) further confirms resilience as a salutogenic construct intrinsically linked to mental health and quality of life.

Operationally, resilience plays a central role in family functioning, serving as a buffer against daily stressors and enhancing overall family wellbeing (Cobos-Sánchez et al., 2022). Parents with strong self-confidence and self-efficacy are better able to manage relational tensions, maintain emotional equilibrium, and implement effective problem-solving strategies (Twiselton et al., 2020). Empirical findings also show that parental resilience facilitates multi-perspective appraisal of challenges, thereby improving conflict-resolution processes within the family (Siahpoosh and GolestaniBakht, 2020).

Children’s emotional competence represents the third construct in this study and is operationalized through two key dimensions. The first, Perception, Understanding, and Expression of One’s Emotions (PU_ones), captures children’s ability to recognize, interpret, and express their internal emotional states. The second dimension, Understanding and Expression of Others’ Emotions (PU_Others), reflects children’s capacity to identify and respond to the emotional experiences of others. Together, these two dimensions provide a comprehensive account of intrapersonal and interpersonal emotional functioning (Di Lorenzo et al., 2019; Rosaria et al., 2019; Kurdi, 2023). Research demonstrates that children who develop competence in these domains engage in social interactions with greater awareness and empathy, which strengthens family relationships and contributes to overall emotional wellbeing.

Taken together, the three constructs form a coherent developmental chain within family systems. Parental wisdom, grounded in cognitive, reflective, and affective capacities, provides the foundational interpretive and regulatory framework through which parents understand and navigate family life. Psychological resilience—expressed through self-confidence and self-efficacy—builds upon this foundation by enabling parents to maintain adaptive functioning and emotional stability when faced with stress. In this sense, resilience operationalizes wisdom: wise reasoning guides *how* parents evaluate situations, while resilience determines how effectively they cope with and respond to them.

These two parental resources jointly create the emotional and relational environment that shapes children’s emotional competence, particularly in the domains of PU_ones and PU_Others. Parents who reason wisely and respond resiliently model emotional clarity, empathy, and constructive coping strategies, thereby fostering children’s ability to perceive, understand, and express emotions in themselves and others. Thus, the constructs are not isolated; rather, they build sequentially—parental wisdom informs resilient parental behavior, and together they cultivate the developmental conditions necessary for children’s emotional competence to flourish.

### Theoretical models and synergistic interactions

Theoretical models and empirical findings converge in demonstrating emotional competence’s protective functions: Olson et al. (2015) documented that elevated emotional competence attenuates psychological threat responses and enhances stress coping capabilities. Schneider et al. (2023) revealed positive correlations between emotional competence and psychological resilience, confirming that advanced emotional processing abilities facilitate more effective life challenge management. Saarni’s (1999) seminal work identified core emotional competence skills—including emotion differentiation, control, and regulation—as essential for maintaining balanced family relationships. Studies further demonstrate that children developing in family environments fostering emotional competence acquire advanced socioemotional abilities enhancing positive social interactions (Suryanarayana, 2022), whereas deficits in children’s emotional competence associate with behavioral and social problems affecting environmental interactions (Havighurst et al., 2004).

### Synergistic interactions: the wisdom-resilience-emotional competence nexus

Emerging evidence suggests that these constructs do not operate in isolation but rather function synergistically within family systems. Research demonstrates that wisdom-resilience interaction facilitates family adaptation to adversity (Peixoto et al., 2023). Kashdan and Rottenberg (2010) elucidated that individuals manifesting both high wisdom and resilience exhibit enhanced challenge-overcoming capacity, promoting family adaptation and constructing more stable, stress-resistant family units (Moreira and Canavarro, 2020).

Recent investigations indicate that the tripartite integration of parental wisdom, resilience, and children’s emotional competence amplifies family wellbeing outcomes. Golestanibakht et al. (2022) found that individuals possessing these capabilities demonstrate superior family challenge management, enhancing psychological health and generating stable family environments (Azadipour and Shoghi, 2023). Zare Mazloom et al. (2024) further documented that such individuals exhibit elevated capacity to confront life challenges using mutual understanding-promoting strategies, yielding greater family cohesion. This three-way interaction strengthens families’ crisis-overcoming abilities and long-term stability achievement (Kashdan and Rottenberg, 2010).

Additionally, wisdom-emotional competence interaction enhances interfamilial emotional communication. Schneider et al. (2023) demonstrated that parental wisdom facilitates empathy-based decision-making, augmenting family emotional communication, while children’s emotional competence—as perceived and potentially modeled by parents—improves expression modalities promoting family harmony (Saarni, 1999). Wise parents consider the long-term consequences of their child-rearing decisions, and their ability to perceive and foster children’s emotional competence facilitates communication that is attuned to children’s affective states, thereby contributing to more balanced and harmonious family environments.

Similarly, the interaction between parental resilience and children’s emotional competence plays in enhancing family life quality. Sharma and Shreya (2024) noted that the development of children’s emotional competence is fundamentally shaped by the quality of the parent-child relationship, which forms the basis for healthy individual growth and long term emotional development trajectories. Positive family interactions, supported by parents’ psychological resilience, strengthen children’s and adolescents’ capacities to construct positive self-concepts and adaptive perceptions of their environment. Family relationships grounded in understanding, emotional balance, and reflective communication foster children’s ability to adapt to stress and overcome crises with flexibility. Collectively, family systems that cultivate wisdom, emotional competence, and resilience create sustainable environments that promote long-term psychological and social wellbeing.

### The Saudi Arabian context: cultural imperatives and vision 2030 alignment

Within the Kingdom of Saudi Arabia, the imperative to develop parental psychological resources such as wisdom and resilience—alongside the cultivation of children’s emotional competence—assumes heightened significance within the national strategic framework of Vision 2030. This transformation agenda explicitly prioritizes human capital development, social cohesion enhancement, and the strengthening of family stability as foundational pillars for sustainable national progress (Alqahtani, 2020; Alenezi et al., 2024). Vision 2030s Quality of Life Program specifically targets the creation of resilient families and the empowerment of individuals with the psychological and socio-economic competencies necessary to navigate rapid societal transformations associated with economic diversification and cultural modernization (Alenezi et al., 2024).

Several specific family challenges and societal shifts underscore the urgency of examining parental wisdom, resilience, and children’s emotional competence in the Saudi context. One of such is that traditional extended family systems are increasingly giving way to nuclear households, altering caregiving patterns, intergenerational support, and socialization practices. These changes can reduce informal familial support, increasing the demands on parents to provide both emotional guidance and adaptive problem-solving skills. Also, increasing maternal workforce participation, combined with evolving paternal responsibilities, creates new pressures on parental time and emotional resources, necessitating higher levels of psychological resilience and wise decision-making to maintain family cohesion (Alqahtani, 2020). Another challenge is their educational and socioemotional reform. Saudi schools are incorporating socioemotional learning into curricula, reflecting national priorities to foster emotional intelligence and interpersonal competence. Parents must adapt their guidance strategies to complement these reforms, requiring effective modeling of emotional competence and resilience. Lastly, Saudi norms emphasize familial interdependence, collective welfare, and respect for hierarchical and intergenerational obligations. These cultural values shape the manifestation of wisdom, resilience, and emotional competence, influencing how parents interact with children and how children develop socioemotional skills. For instance, affective and reflective dimensions of parental wisdom may play a heightened role in navigating culturally embedded expectations of family harmony and decision-making.

The network analysis approach is particularly useful in this context as it allows identification of critical relational pathways linking parental psychological resources with children’s emotional competence, revealing which elements (e.g., self-efficacy, cognitive wisdom, emotion regulation) are central in supporting family adaptation and child development. By modeling interconnected subdimensions rather than aggregate scores, the approach captures culturally specific mechanisms—such as how collective-family norms or intergenerational obligations modulate emotional competence transmission—that traditional regression approaches may obscure. Network models can highlight leverage points for policy and interventions, enabling evidence-based guidance for family education programs, counseling initiatives, and public strategies aligned with Vision 2030. For example, identifying the central nodes in the wisdom-resilience-emotional competence network may inform targeted parent training programs that enhance children’s socioemotional outcomes while respecting Saudi cultural norms.

Taken together, examining parental wisdom, resilience, and children’s emotional competence within Saudi families is not only theoretically meaningful but also practically imperative. By aligning psychological constructs with Vision 2030 objectives, this study provides a culturally grounded rationale for intervention, identifying actionable pathways through which national goals—such as building resilient, competent families—can be achieved.

### Research gaps and the network analysis imperative

Despite substantial theoretical and empirical attention to wisdom, psychological resilience, and emotional competence as individual constructs, critical gaps remain in understanding their structural interrelationships within family systems, particularly in non-Western contexts. Although prior studies have examined these constructs in pairwise relationships (e.g., wisdom-resilience: Peixoto et al., 2023; resilience-emotional competence: Schneider et al., 2023), no research has investigated them as a dynamic, interconnected network system where all constructs simultaneously influence one another within parent-child dyads. Traditional variable-centered analyses—such as linear regression or structural equation modeling—require the imposition of a priori directional assumptions and may obscure the complex, bidirectional and recursive interdependencies that characterize real-world family psychological systems (Borsboom et al., 2021).

Second, existing research has predominantly employed Western samples, with minimal investigation of how cultural contexts—particularly collectivist, family-centered Arab societies like Saudi Arabia—may shape the manifestation and interrelation of these psychological constructs. The cultural specificity question remains empirically unresolved: Do wisdom, resilience, and emotional competence interact differently in family systems characterized by distinct cultural norms, family structures, and parenting practices?

Third, methodological limitations in prior research include reliance on total scale scores that aggregate multidimensional constructs, that potential mask granular relationships between specific subdimensions (e.g., cognitive vs. reflective wisdom components; emotion recognition vs. regulation aspects of emotional competence). Ardelt et al. (2018) explicitly recommended employing measurement instruments that capture the cognitive, reflective, and affective components of wisdom, alongside key resilience factors and emotional competence dimensions to achieve robust construct representation; however, studies that integrate these recommendations within comprehensive network frameworks remain scarce.

Fourth, from an applied perspective, identifying which specific elements within the wisdom-resilience-emotional competence system function as central nodes or critical bridges holds direct implications for intervention optimization. When specific components demonstrate disproportionate influence within the network (high centrality), targeting these leverage points in family interventions may yield cascading benefits across the entire system; however, such precision is unattainable without network-based analyses that identify structural importance metrics.

Finally, the stability and replicability of identified relationships—a fundamental concern in psychological science—requires rigorous evaluation through resampling techniques that remain underutilized in family psychology research. Establishing whether observed network structures remain robust under varied sampling conditions is essential for generalizability claims and evidence-based practice recommendations.

The present study addresses these gaps by employing psychometric network analysis to examine the structural relationships among parental wisdom dimensions, psychological resilience components, and parent-perceived children’s emotional competence within Saudi Arabian families. This approach offers three critical advantages: (1) it provides a theoretical, data-driven identification of direct associations between variables without imposing predetermined structural models (Marsman et al., 2018); (2) it quantifies each variable’s centrality—its relative importance and influence within the network—enabling identification of intervention leverage points (Epskamp et al., 2018; Letina et al., 2019); and (3) it reveals bridging relationships that connect distinct psychological domains, illuminating potential mechanisms underlying family adaptation and child development (Fonseca-Pedrero et al., 2018).

For Saudi family policy and intervention contexts specifically, network analysis offers actionable insights: by identifying which parental psychological resources most strongly associate with children’s emotional competence, and which specific subdimensions function as critical bridges, findings can inform targeted parental education programs, family counseling protocols, and social service resource allocation aligned with Vision 2030s family strengthening objectives. Understanding these network structures within the Saudi cultural context may reveal culturally specific mechanisms requiring tailored intervention approaches rather than direct Western model translations.

### Network analysis framework: conceptual and methodological foundations

To clarify the analytical approach underlying this study’s research questions, a brief explication of network analysis principles is warranted. Network analysis, as applied in psychometric contexts, conceptualizes psychological phenomena as complex systems comprising interconnected elements—nodes (variables or constructs)—linked by edges (statistical associations representing potential causal or mutually reinforcing relationships) (Epskamp et al., 2018; Borsboom and Cramer, 2013). Unlike latent variable models that treat observed indicators as reflections of underlying common causes, network models assume indicators exist and co-occur because they directly influence one another through mutually reinforcing processes (Borsboom et al., 2021). This ontological shift is particularly relevant for family psychology, where wisdom, resilience, and emotional competence may reciprocally influence each other through lived family interactions rather than merely reflecting an underlying “family functioning” latent variable. Key network metrics include:

Edges (connections): Statistical associations between nodes after controlling for all other network variables, representing direct relationships rather than spurious correlations induced by shared causes. Strong edges indicate robust direct associations; edge patterns reveal the network’s structural architecture.

Bridges: Edges connecting distinct communities or clusters within a network. In family contexts, bridge edges linking parental resources (wisdom, resilience) with child outcomes (emotional competence) are theoretically and practically significant, as they represent potential transmission mechanisms through which parental characteristics influence child development.

Centrality indices: Quantitative metrics assessing each node’s importance within the network structure:

Expected Influence: The sum of a node’s edge weights, indicating how strongly a variable connects to others in the network. High expected influence suggests a variable is a core hub whose changes may propagate throughout the system.

Betweenness: The frequency with which a node lies on shortest paths between other nodes, indicating bridging potential.

Closeness: The inverse of a node’s average distance to all other nodes, reflecting how quickly changes might spread from that node.

Network stability: The robustness of centrality estimates and edge weights under case-dropping bootstrap procedures. Stability coefficients (CS-coefficients) quantify the proportion of the sample that can be dropped while maintaining correlations above 0.70 between original and resampled centrality values; CS > 0.50 indicates acceptable stability (Epskamp et al., 2018).

Network models do not presuppose a priori theoretical structures, making them valuable for identifying alternative mechanisms and exploratory hypothesis generation (Marsman et al., 2018). This atheoretical, data-driven character is particularly advantageous when investigating understudied cultural contexts or complex multivariable systems where theoretical guidance is limited. Moreover, network analysis enables granular examination of relationships among subdimensions of multifaceted constructs—an essential feature for capturing the cognitive, reflective, and affective components of wisdom; resilience factors; and emotional competence dimensions with maximal fidelity (Ardelt et al., 2018; Grossmann et al., 2020; Webster et al., 2018).

In family research contexts, network analysis illuminates how parental psychological resources and child developmental outcomes interconnect within dynamic family systems influenced by bidirectional parent-child interactions (Fonseca-Pedrero et al., 2018). This approach can reveal whether wisdom functions as a central node integrating resilience and emotional competence, how different construct components interact across family dynamics, and which specific pathways might serve as optimal intervention targets (Chen et al., 2019; Ferrari and Weststrate, 2013). For Saudi families navigating societal transitions, identifying these network structures provides empirically grounded guidance for developing culturally responsive family support programs.

### Present study aims

Despite extensive research demonstrating the importance of parental wisdom and psychological resilience in shaping children’s socioemotional outcomes, limited work has examined how these constructs collectively influence children’s emotional competence within a coherent, theoretically grounded framework. Moreover, prior studies often rely on variable-centered approaches that assume linear, unidirectional relationships, potentially obscuring the complex, multidimensional interactions inherent in family systems. To address these gaps, the present study employs psychometric network analysis, which allows simultaneous examination of interconnected parental and child constructs without imposing restrictive structural assumptions, while maintaining alignment with theoretical expectations regarding family functioning.

Guided by theories on wisdom, resilience, and emotional competence, this study conceptualizes parental wisdom as comprising cognitive, reflective, and affective domains, parental resilience as consisting of self-confidence and self-efficacy, and children’s emotional competence as involving intrapersonal (PU_ones) and interpersonal (PU_Others) dimensions. The integration of these constructs reflects a theoretically informed developmental chain in which:

1. Parental wisdom provides the interpretive and regulatory framework for navigating family life;
2. Parental resilience enables adaptive coping and emotional stability under stress; and
3. Children’s emotional competence emerges through parental modeling, guidance, and relational support.

The study leverages network analysis to examine these constructs as a complex, dynamic system, allowing identification of core nodes, bridging elements, and direct associations that reflect theoretically expected mechanisms of intergenerational influence. This study aims to provide a theoretically anchored, data-driven understanding of how parental psychological resources relate to children’s emotional competence in Saudi families, with implications for family interventions and parental education programs aligned with Vision 2030 objectives. Specifically, it addresses the following research questions:

*RQ1*: Which edges, theoretically informed by prior research, function as bridges connecting parental wisdom, psychological resilience, and children’s emotional competence?

This question examines mechanisms of intergenerational influence, testing whether theoretically expected pathways—such as affective wisdom and parental self-efficacy facilitating children’s intrapersonal and interpersonal emotional competence—emerge as bridge connections within the network.

*RQ2:* Which psychological constructs demonstrate the highest centrality within the family network, consistent with theory-informed expectations?

This question assesses whether specific dimensions of parental wisdom or resilience, or aspects of children’s emotional competence, occupy central positions in the network, reflecting their theorized importance as leverage points for promoting adaptive family functioning.

*RQ3*: How robust and stable are these network relationships under resampling conditions, and do these patterns support theoretical expectations?

This question evaluates the replicability and stability of network edges, centrality, and bridging metrics, confirming whether the observed structure aligns with theory-informed hypotheses regarding the relationships among parental wisdom, resilience, and children’s emotional competence.

By integrating theoretical grounding with the exploratory, data-driven advantages of network analysis, this study provides a nuanced perspective on how parental resources support children’s socioemotional development in the Saudi context. Findings are expected to identify critical nodes and pathways that can inform culturally sensitive family interventions, parent education programs, and policy initiatives aimed at enhancing family wellbeing and supporting children’s emotional competence during formative developmental periods.

## Materials and methods

### Participants

#### Sample size and composition

The final analyzed sample included 252 parents after excluding 18 incomplete responses, resulting in a completion rate of 93.3%. The sample consisted of 95 fathers (37.7%) and 157 mothers (62.3%), reflecting a distribution reasonably representative of primary caregiving patterns in Saudi families, where mothers typically assume greater direct parenting responsibilities, while fathers maintain active but comparatively less direct involvement. Participants were recruited through a multi-channel approach over a 3-month data collection period. The sampling frame was constructed to capture geographic diversity spanning major urban centers (Riyadh, Jeddah, Dammam, Makkah) and regional cities across the Central, Western, Eastern, Southern, and Northern provinces of Saudi Arabia.

Although an a priori power analysis specific to network models was not conducted, the selected sample size (*N* = 252) was considered acceptable given the relatively simple network structure comprising seven nodes. Simulation-based studies in network psychometrics suggest that smaller networks (fewer than 10 nodes) can yield reasonably interpretable GLASSO estimates with sample sizes in the range of 200–300, particularly when the primary focus is on edge estimation and strength centrality rather than more unstable indices such as betweenness.

Accordingly, the present sample size was deemed sufficient to provide an initial exploratory estimation of the network structure, while recognizing that larger samples (e.g., *N* > 400–500) are generally recommended to achieve higher stability coefficients (CS ≥ 0.50), especially for centrality metrics beyond strength.

Recruitment strategies included:

1. Digital recruitment channels: Online surveys distributed via social media platforms (Twitter/X, WhatsApp family groups, Telegram channels) targeting Saudi parenting communities and educational forums. Survey links were shared through parenting-focused Saudi social media accounts and verified community groups.
2. Community-based recruitment: Collaboration with community centers (marākiz mujtama’iyya), educational institutions (public and private schools), and family service centers in participating cities. Informational flyers and digital announcements were posted with institutional permissions.
3. Snowball referral component: Participating parents were invited to share the survey link with other eligible parents in their social networks, broadening geographic and demographic reach.

All recruitment materials clearly stated the study purpose, voluntary nature of participation, time commitment (approximately 20–25 min), and anonymity assurances.

Inclusion and exclusion criteria: Inclusion criteria required that participants:

1. Be biological or adoptive parents currently residing in Saudi Arabia
2. Have at least one child aged 5–20 years living in the household at the time of data collection
3. Possess sufficient Arabic literacy to complete self-report questionnaires independently
4. Provide informed consent to participate

Exclusion criteria eliminated participants who:

1. Reported clinically diagnosed severe mental health disorders (e.g., schizophrenia, bipolar disorder, major depressive disorder requiring hospitalization) that might compromise response validity
2. Had children with formally diagnosed neurodevelopmental disorders (e.g., autism spectrum disorder, severe intellectual disability) requiring specialized care, as this may fundamentally alter parent-child emotional dynamics beyond the scope of the current study
3. Were non-Saudi residents or temporary visitors without established family residence in Saudi Arabia
4. Completed fewer than 80% of survey items (partial completions)
5. These criteria were designed to capture typical parent-child dyads in Saudi family contexts while maintaining sample homogeneity for interpretable network analysis.

### Parental demographics

1. Age distribution: Participants ranged from 20 to 69 years. The modal age group was 40–49 years (45.6%, *n* = 115), followed by 30–39 years (31.3%, *n* = 79), 50–59 years (14.3%, *n* = 36), 20–29 years (7.1%, n = 18), and 60–69 years (1.6%, *n* = 4).
2. Marital duration: Nearly half of participants (49.2%, *n* = 124) reported 11–20 years of marriage, 27.4% (*n* = 69) had been married 21–30 years, 11.9% (*n* = 30) had been married for less than 10 years, and 11.5% (*n* = 29) had been married more than 30 years.
3. Geographic distribution: Participants were drawn from multiple regions of Saudi Arabia: Central Region, 38%; Western Region, 27%; Eastern Region, 20%; Southern/Northern Regions, 15%].
4. Educational background: 45% held bachelor’s degrees, 28%, secondary education; 18%, postgraduate degrees, 9%, intermediate or lower.

### Child demographics

1. Each parent provided information regarding one target child, yielding data on 252 children comprising 157 males (62.3%) and 95 females (37.7%).
2. Age distribution: The largest child age group was 10–15 years (42.5%, *n* = 107), followed by under 10 years (40.1%, *n* = 101), with 17.5% (*n* = 44) aged 16–20 years. This distribution ensured representation across critical developmental periods (middle childhood, early adolescence, late adolescence). Table 1 presents comprehensive demographic characteristics of both parent and child samples.

**TABLE 1:** Participant demographics.

Variable	Category	Frequency	Percent (%)	Valid percent (%)	Cumulative percent (%)
Parent type	(Father)	95	37.7	37.7	37.7
	(Mother)	157	62.3	62.3	100
Parent age	20–29 years	9	3.6	3.6	3.6
	30–39 years	79	31.3	31.3	34.9
	40–49 years	115	45.6	45.6	80.6
	50–59 years	39	15.5	15.5	96
	60–69 years	10	4	4	100
Marriage duration	Less than 10 years	30	11.9	11.9	11.9
	More than 30 years	29	11.5	11.5	23.4
	11–20 years	124	49.2	49.2	72.6
	21–30 years	69	27.4	27.4	100
Child gender	Female	95	37.7	37.7	37.7
	Male	157	62.3	62.3	100
Child age	Less than 10 years	101	40.1	40.1	40.1
	10–15 years	107	42.5	42.5	82.5
	16–20 years	44	17.5	17.5	100
	Total	252	100	100	–

### Rationale for sample composition

The study’s diverse sample—encompassing parents spanning four decades of adulthood, varied marital durations, and children across three developmental stages (childhood, early adolescence, mid-to-late adolescence)—was intentionally designed to capture heterogeneity in family life cycle stages and parent-child relational dynamics. This diversity enhances the generalizability of identified network structures across typical Saudi family configurations while providing sufficient variability in constructs of interest (wisdom accumulation across parental ages, mothers, despite numerical imbalance reflecting differential recruitment accessibility, permits preliminary exploration resilience tested through varied marital durations, emotional competence across child developmental periods) to enable robust network estimation. The inclusion of both fathers and of potential gender-specific network patterns in supplementary analyses.

### Procedure

#### Ethical declaration

This study adhered to ethical principles and guidelines for research involving human participants. Informed consent was obtained in written form from all participants during the period of December 2024 to February 2025. Participants were informed about the purpose of the study, the procedures involved, their right to withdraw at any time without penalty, and the confidentiality and anonymity of their responses were ensured throughout the research process.

#### Ethical approval

This research received ethical approval from the Institutional Review Board at King Faisal University. The University Research Ethics Committee approved the study on December 12, 2024, under reference number KFU-REC-2024-DEC-ETHICS2961. All research procedures followed established ethical guidelines to ensure the rights, safety, and wellbeing of participants. The approval process reflects the researchers’ full commitment to conducting ethically sound and scientifically valid research.

#### Study design and sampling framework

This cross-sectional study employed a simple random sampling technique to ensure representative coverage of the target population across multiple regions of the Kingdom of Saudi Arabia. The sampling approach aimed to minimize selection bias by providing equal opportunity for participation among eligible parents across diverse geographic locations and demographic backgrounds.

#### Data collection procedure

Survey Platform and Format: Data collection occurred via secure online survey Google Forms with security settings accessible through distributed links. The survey interface was presented entirely in Arabic, utilizing validated Arabic translations of all measurement instruments.

#### Informed consent process

Prior to survey access, potential participants encountered a detailed digital informed consent page explaining:

1. Study purpose (investigating relationships among parental psychological characteristics and children’s emotional development)
2. Procedures (completion of three validated psychological questionnaires)
3. Voluntary participation and withdrawal rights without penalty
4. Data anonymity (no personally identifiable information collected) and confidentiality protections
5. Estimated completion time (20–25 min)
6. Researcher contact information for questions or concerns
7. IRB approval reference number (KFU-REC-2024-DEC-ETHICS2961)

Participants indicated informed consent by selecting “I agree to participate” before proceeding to survey items. Only consenting individuals accessed the measurement instruments. Given that the study relied on self-report measures—particularly regarding potentially sensitive parenting perceptions and family dynamics—oral informed consent was deemed appropriate and ethically sufficient for this minimal-risk online survey research.

#### Survey structure and administration

The online survey presented instruments in the following fixed order to maintain standardization:

1. Demographic information questionnaire (parent and target child characteristics)
2. 12-Item Abbreviated Three-Dimensional Wisdom Scale (3D-WS-12)
3. Resilience Evaluation Scale (RES)
4. Parental Assessment Questionnaire for Children’s Emotional Competence (URPEKD)

All items were mandatory except demographic questions coded as sensitive (e.g., income), minimizing missing data. Progress indicators displayed completion percentage to encourage survey completion. The survey required approximately 20–25 minutes to complete, with no time limits imposed to allow thoughtful responding.

*Quality control measures*: To ensure response validity and data quality:

1. Multiple submission prevention: Survey platform settings prevented multiple submissions from the same device/IP address, ensuring each participant contributed only once.
2. Attention checks: Two attention-check items were embedded (e.g., “Please select ‘strongly agree’ for this item”) to identify careless or inattentive responding. Participants failing both attention checks were excluded from analysis.
3. Completion time monitoring: Survey completion times were monitored. Surveys completed in less than 8 min (suggesting insufficient engagement or random responding) were flagged for review and excluded if response patterns indicated invalidity.
4. Response pattern analysis: Data screening identified straight-lining (identical responses across all scale items within a measure), which suggests satisficing behavior. Cases exhibiting straight-lining across multiple measures (*n* = 5) were excluded from the final dataset.
5. Missing data handling: Participants who completed fewer than 80% of survey items were automatically excluded. The mandatory response feature minimized item-level missing data for included participants.

*Anonymity and confidentiality protections*: Participants received explicit assurances regarding data protection:

1. No personally identifiable information: The survey did not collect names, contact details, national identification numbers, or any information that could directly identify participants.
2. IP address protection: Survey platform settings disabled IP address logging to prevent traceability.
3. Secure data storage: Collected data were stored on password-protected, encrypted servers accessible only to research team members.
4. Aggregate reporting: Only group-level aggregated data would be reported in publications and presentations; individual responses would never be disclosed.
5. Third-party restrictions: Individual responses would never be shared with third parties, including governmental agencies, educational institutions, or family members.

These comprehensive protections aimed to minimize social desirability bias and encourage honest responding, particularly concerning sensitive topics such as parenting efficacy, family conflicts, or children’s emotional difficulties. Participants were explicitly informed that their responses would not be shared with spouses, children, or any other family members, promoting candid self-assessment.

### Measurements

#### The Wisdom Scale

The 12-Item Abbreviated Three-Dimensional Wisdom Scale (3D-WS-12) by Thomas et al. (2017) is a psychometric tool designed to measure wisdom across three key dimensions: cognitive, reflective, and affective (compassionate). The scale consists of 12 items, with each dimension represented by four items. The cognitive dimension assesses an individual’s ability to understand life’s complexities and embrace uncertainty. The reflective dimension evaluates self-awareness and openness to different perspectives. Lastly, the affective dimension measures empathy and emotional engagement. Responses are rated on a 5-point Likert scale ranging from 1 (strongly disagree) to 5 (strongly agree), with total scores ranging from 12 to 60, where higher scores indicate greater wisdom. The scale has demonstrated good internal consistency, with Cronbach’s alpha values generally exceeding 0.70, and strong construct validity, correlating well with measures of emotional intelligence, perspective-taking, and life satisfaction. Due to its brevity and reliability, the 3D-WS-12 serves as a valuable instrument for research and clinical applications in assessing wisdom-related traits.

The current research translated the scale, achieving a McDonald’s omega reliability coefficient of 0.728, which indicates a good level of internal consistency and demonstrates the reliability of the translated measurement instrument across the study’s sample.

The Resilience Evaluation Scale (RES) was developed by van der Meer et al. (2018). It was designed to assess psychological resilience and is available in both English and Dutch. The scale consists of nine items, measuring two key components of resilience: self-confidence and self-efficacy. Each component is represented by three items. The RES uses a 5-point Likert scale, typically ranging from 1 (strongly disagree) to 5 (strongly agree). The total score is calculated by summing all item responses, with a minimum possible score of 9 (indicating low resilience) and a maximum possible score of 45 (indicating high resilience).

Psychometric evaluation of the RES has shown good internal consistency, with Cronbach’s alpha values above 0.80 for the overall scale. The scale also demonstrates strong construct validity, as it correlates well with other established resilience measures and psychological wellbeing indicators. Given its brevity and strong psychometric properties, the RES is a reliable tool for assessing resilience in both research and clinical contexts. The current research translated the scale, achieving a McDonald’s omega reliability coefficient of 0.868, which indicates a good level of internal consistency and demonstrates the reliability of the translated measurement instrument across the study’s sample.

The Parental Assessment Questionnaire for Children’s Emotional Competence (URPEKD) by Mikèević-Milković et al. (2020) was developed and validated to assess children’s emotional competence from a parental perspective. The scale evaluates key aspects of emotional competence, such as emotional expression, emotion regulation, and social-emotional understanding. The questionnaire consists of multiple items rated on a Likert scale, where parents indicate the extent to which they observe specific emotional competencies in their children. The scale consists of two dimensions. The first dimension is titled “The Perception, Understanding, and Expression of One’s Emotions,” and it is denoted in the study as PU_ones. The second dimension is titled “Understanding and Expression of Others’ Emotions,” and it is represented in the study by the symbol PU_Others.

The total score is obtained by summing the responses, with higher scores indicating greater emotional competence and lower scores reflecting potential difficulties in emotional development. Psychometric analysis on the URPEKD has demonstrated good reliability, with Cronbach’s alpha values exceeding 0.80, indicating strong internal consistency. Additionally, the scale has shown good construct validity, as it correlates well with other established measures of emotional competence and social-emotional development. Given its solid psychometric properties, the URPEKD is a valuable tool for research and practical applications in developmental psychology and parental assessments. The current research translated the scale and examined its reliability using McDonald’s omega coefficient. The results confirmed satisfactory reliability for both the total score and the two dimensions of the scale. The McDonald’s omega coefficients were 0.942 for the total score, 0.875 for the first dimension (PU_ones), and 0.890 for the second dimension (PU_Others), indicating a high level of internal consistency and demonstrates the reliability of the translated measurement instrument across the study’s sample.

### Statistical analysis

Data analysis was conducted using SPSS 28 and R (version 4.1.2). Descriptive statistics, Pearson correlations, and McDonald’s omega were computed in SPSS to assess reliability and the relationships between variables. Additionally, network analysis was performed using the R package bootnet (version 1.4.3) to examine the structural relationships between study variables.

A Graphical Least Absolute Shrinkage and Selection Operator (GLASSO) model was estimated using the GLASSO algorithm, combined with the extended Bayesian information criterion (EBICglasso) for optimal model selection (Friedman et al., 2008). The tuning parameter was set to 0.5 to ensure a parsimonious and interpretable network structure. Edges were used to depict connections between variables, with their thickness representing the strength of associations. Blue edges indicated positive correlations, while red edges represented negative correlations. Network centrality was assessed through three key metrics: betweenness (degree of connectivity), closeness (distance centrality), and strength (degree centrality) (Opsahl et al., 2010). Centrality values were reported as standardized z-scores.

To assess network stability and accuracy, a bootstrap procedure with 1,000 resamples was applied using the bootnet package. Bootstrapped 95% confidence intervals were used to estimate edge–weight accuracy, where narrower intervals indicated higher precision. The case-dropping bootstrap method was employed to evaluate node stability, with results visualized graphically. The correlation stability coefficient (CS-coefficient) was calculated, with values above 0.5 indicating strong stability and values below 0.25 suggesting weak interpretability (Epskamp et al., 2018).

Additionally, the small-worldness index (SWI) was computed to determine whether the network exhibited small-world properties. The SWI formula incorporated both the average shortest path length (L) and the global clustering coefficient (C) compared to an Erdös–Rényi random network with the same number of nodes and connections (Watts and Strogatz, 1998). An SWI > 1 indicated a highly interconnected network with strong overall connections and efficient information transfer.

## Results

### Descriptive statistics and correlation analyses

Table 2 presents the descriptive statistics and Pearson correlations for all study variables. PU_ones (Perception, Understanding, and Expression of One’s Emotions) and PU_Others (Perception, Understanding, and Expression of Others’ Emotions) are subscales of the Parental Assessment Questionnaire for Children’s Emotional Competence (URPEKD; Mikèević-Milković et al., 2020). In the present sample, PU_ones demonstrated high internal consistency (McDonald’s ω = 0.875), and PU_Others also showed excellent reliability (ω = 0.890), confirming the psychometric validity of these subscales for Saudi parents.

**TABLE 2:** Descriptive statistics and correlation analyses.

Variable	Mean	SD	Cognitive	Reflective	Affective	Self_conf	PU_ones	PU_Others	Wisdom	Resilience	Emotional
Cognitive	10.27	3.339	1	1	1	1	1	1	1	1	1
Reflective	13.69	2.605	0.335**								
Affective	13.13	2.285	0.396**	0.370**							
Self_conf	20.35	2.885	−0.165**	−0.103	0.209**						
Efficacy	16.14	2.445	−0.116	−0.125*	0.188**	0.744**					
PU_ones	30.04	6.038	−0.043	−0.051	0.132*	0.454**					
PU_Others	21.57	4.090	0.114	0.076	0.203**	0.320**	0.590**				
Wisdom	37.0913	6.27932	0.815**	0.727**	0.728**	−0.054	0.004	0.166**			
Resilience	36.4960	4.98084	−0.152*	−0.121	0.213**	0.945**	0.511**	0.327**	−0.054		
Emotional	51.6111	9.07514	0.023	0.000	0.180**	0.446**	0.931**	0.844**	0.078	0.487**	

* Indicates statistical significance at *p* < 0.05.

** Indicates statistical significance at *p* < 0.01.

Cognitive variable was significantly and positively correlated with reflective (*r* = 0.335, *p* < 0.01), affective (*r* = 0.396, *p* < 0.01), and wisdom (*r* = 0.815, *p* < 0.01), indicating that individuals with higher cognitive abilities tend to exhibit higher reflective thinking, emotional engagement, and wisdom. Self-confidence showed a significant negative correlation with cognitive (*r* = −0.165, *p* < 0.01) but was positively associated with affective (*r* = 0.209, *p* < 0.01) and efficacy (*r* = 0.744, *p* < 0.01), suggesting that self-confidence is more closely linked with emotional and motivational dimensions than with cognitive processing.

Perceived emotional competence, measured via PU_ones and PU_Others, was positively associated with self-confidence (*r* = 0.454, *p* < 0.01; *r* = 0.320, *p* < 0.01, respectively) and affective engagement (*r* = 0.931, *p* < 0.01; *r* = 0.844, *p* < 0.01, respectively), indicating that parents who perceived higher emotional competence in their children tended to report greater self-confidence and emotional involvement.. Overall wisdom was strongly correlated with its subdimensions—cognitive (*r* = 0.815, *p* < 0.01), reflective (*r* = 0.727, *p* < 0.01), and affective (*r* = 0.728, *p* < 0.01)—reinforcing the integrative nature of wisdom across intellectual and emotional domains. Resilience exhibited strong positive correlations with self-confidence (*r* = 0.945, *p* < 0.01) and affective engagement (*r* = 0.487, *p* < 0.01), highlighting the role of psychological resources in supporting both adaptive functioning and emotional competence.

Finally, resilience showed a strong positive correlation with self-confidence (*r* = 0.945, *p* < 0.01) and a moderate positive correlation with children’s emotional competence as perceived by parents (PU_ones; *r* = 0.487, *p* < 0.01). This suggests that parents with higher self-confidence tend to be more resilient and perceive their children as demonstrating greater emotional competence. The strong associations among parental resilience, self-confidence, and perceived child emotional competence highlight the interdependence of cognitive and emotional factors in supporting adaptive family functioning.

### Network analysis variables

#### Characteristics of edges

The GLASSO (EBIC) network model revealed a psychometric structure with seven nodes and 14 edges, demonstrating a moderate edge density of 0.667. The edge weights ranged from −0.104 to 0.598, with an average weight of 0.156, indicating varying levels of association between constructs. The strongest connections were observed between self-confidence and self-efficacy (0.6) and between the perception, understanding, and expression of one’s emotions and the understanding and expression of others’ emotions (0.46), highlighting key relationships in the network.

The Walktrap algorithm detected three distinct communities:

1. Wisdom (cognitive, reflective, and affective);
2. Psychological resilience (self-confidence and self-efficacy); and
3. Emotional competence (the perception, understanding, and expression of one’s emotions and the understanding and expression of others’ emotions).

This clustering suggests that cognitive and emotional aspects are functionally distinct yet interconnected. The Louvain algorithm confirmed the multidimensional nature of the network, while the TEFI (Total Entropy Fit Index) score (−2.724) supported the model’s stability. The SWI = 1.028 indicated that the network exhibited small-world properties characterized by high clustering and short path lengths, making it efficient for information transfer.

Correlation stability (CS) analysis revealed a maximum drop proportion of 5.2% to maintain a correlation of 0.7 in at least 95% of the samples, suggesting that the network structure was relatively stable under resampling conditions. However, accuracy could be improved with increased bootstrapping iterations.

The edge betweenness centrality results highlighted the most influential connections within the network, where edges with higher betweenness values serve as key bridges facilitating information transfer between constructs. The strongest edge betweenness was observed between self-confidence and efficacy (10), indicating their pivotal role in connecting different psychological domains. Other significant connections included efficacy and PU_Ones (8), as well as affective and self-confidence (6), suggesting that these relationships are crucial in linking cognitive, affective, and behavioral components. Weaker betweenness values for cognitive-reflective (1) and cognitive-affective (1) suggest these links play a minor role in overall network connectivity.

The shortest path lengths results provide insights into how efficiently information flows between nodes. A shorter path length signifies a stronger and more direct relationship. The closest connections existed between self-confidence and efficacy (1.63) and PU_Ones and PU_Others (2.12), reinforcing the strong psychological interplay between these constructs. In contrast, longer path lengths, such as cognitive to PU_Others (15.83) and reflective to PU_Ones (18.58), indicate that constructs are more distantly related, requiring multiple intermediary steps for interaction.

**FIGURE 1:**
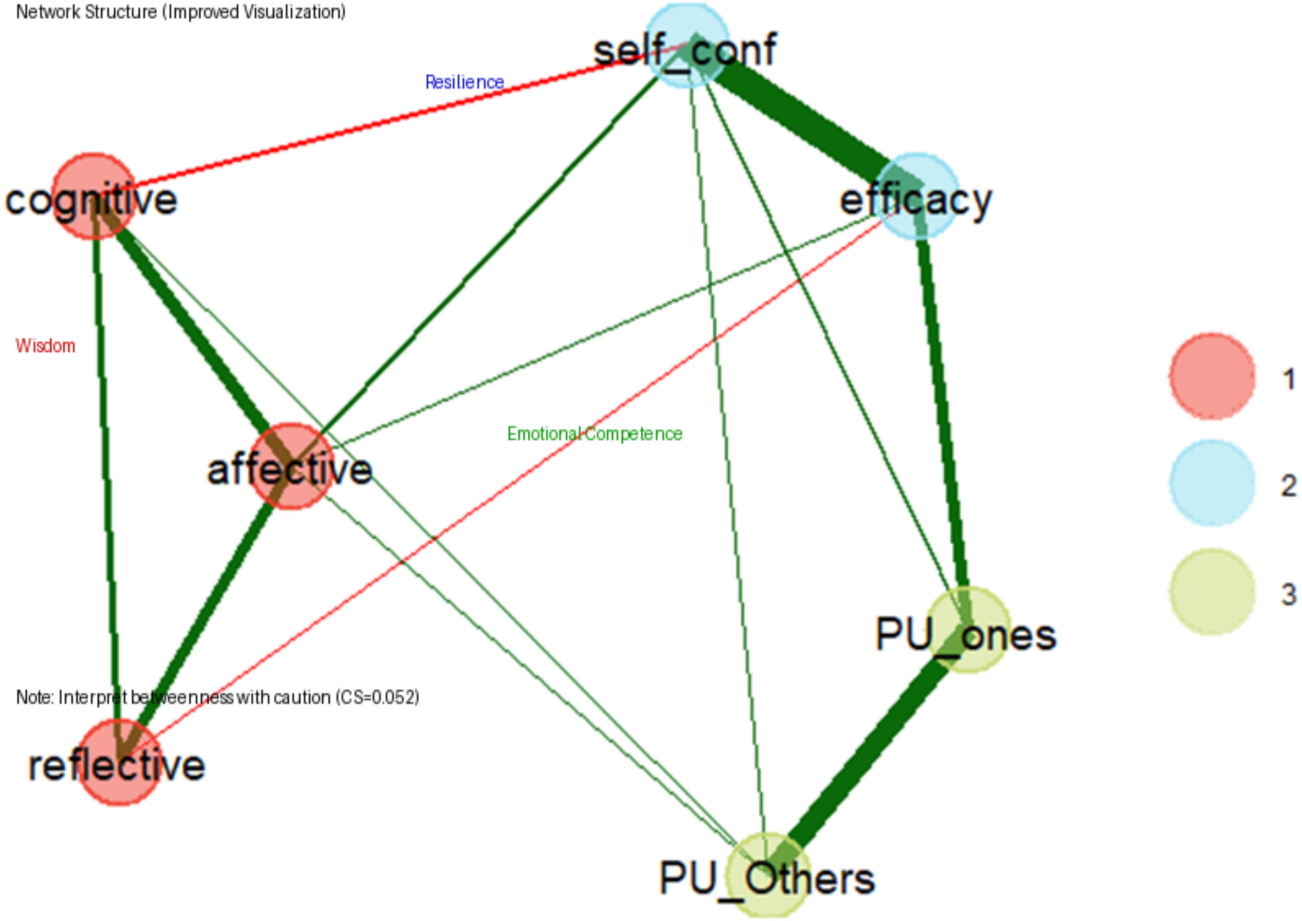
Psychometric network of psychological resilience, wisdom, and emotional competence. Network structure of parental wisdom, psychological resilience, and children’s emotional competence. Nodes represent study variables, grouped into three communities (Wisdom, Resilience, Emotional Competence). Green edges indicate positive associations, while red edges indicate negative associations. Edge thickness reflects the strength of partial correlations. Given the low stability coefficient (CS = 0.052), interpretations of betweenness centrality and bridge roles should be approached with caution.

The psychometric network of psychological resilience, parental wisdom, and emotional competence is shown in Figure 1, suggesting a well-structured and resilient network where psychological resilience and emotional competence play central roles in linking cognitive and affective components. The small-world properties further enhance network efficiency, making it a meaningful representation of the underlying psychological constructs.

#### Characteristics of nodes

**FIGURE 2:**
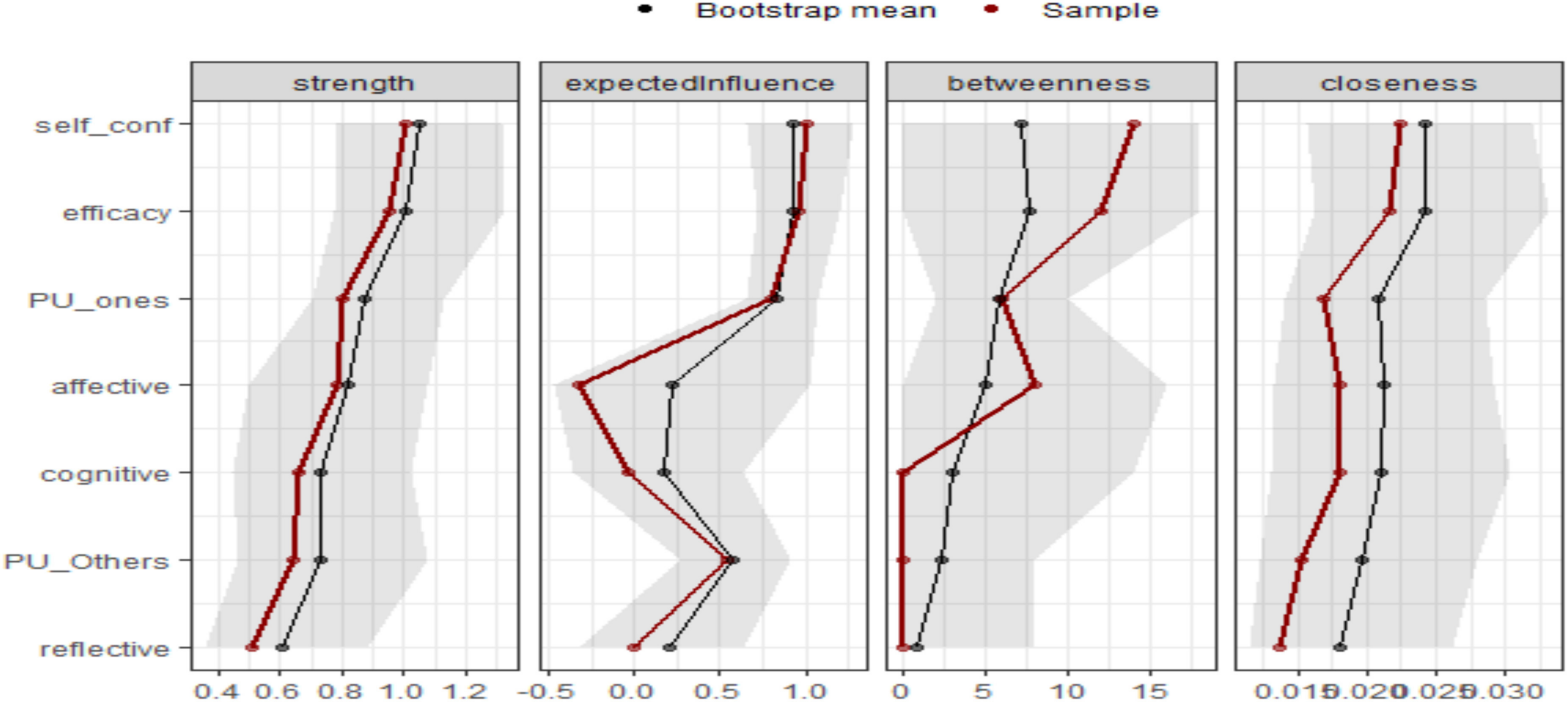
Centrality plots of the EBICglasso network depicting the betweenness, closeness, degree (strength), and expected influence of each node.

Figure 2 and Table 3 show the characteristics of nodes in the psychometric network, which revealed important structural properties of the variables based on centrality measures:

**TABLE 3:** Centrality measures between variables in the network.

Node	Betweenness	Closeness	Strength	Expected influence
Cognitive	0	0.018	0.656	0.398
Reflective	0	0.014	0.510	0.361
Affective	4	0.018	0.781	0.781
Self-confidence	7	0.022	1.008	0.734
Efficacy	6	0.022	0.952	0.818
PU_Ones	3	0.017	0.797	0.797
PU_Others	0	0.015	0.642	0.642

1. Self-confidence and efficacy exhibited the highest strength centrality (1.008 and 0.952, respectively), indicating their crucial roles in the network by having the most substantial direct connections with other variables.
2. Affective, PU_Ones, and efficacy also demonstrated high expected influence, signifying their importance in spreading activation within the network.
3. Betweenness centrality, which represents how often a node acts as a bridge, was highest for self-confidence (7) and efficacy (6), reinforcing their intermediary role in connecting different constructs.
4. Closeness centrality, which reflects the ease of reaching other nodes, was highest for self-confidence (0.022) and efficacy (0.022), suggesting their efficiency in communicating across the network.
5. Cognitive, reflective, and PU_Others had the lowest betweenness (0) and relatively lower strength, indicating a more peripheral role in the network structure.

The bootstrap analysis confirmed the stability of these centrality indices, showing a consistent pattern between the sample estimates (red) and bootstrap means (black). The shaded areas indicate the variability in estimates, with relatively small deviations suggesting reliable network measures.

**FIGURE 3:**
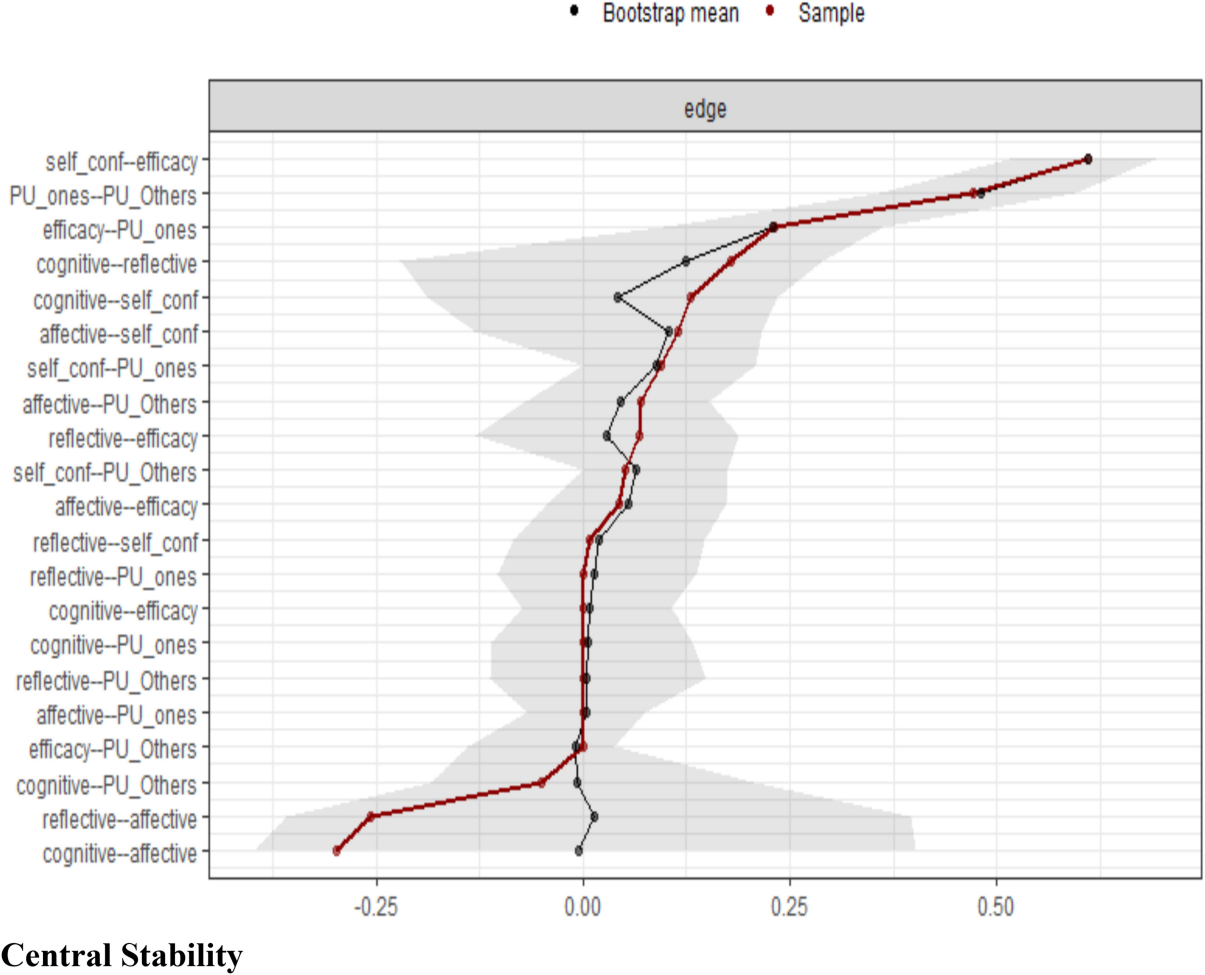
Bootstrapped confidence intervals for edge weights in the psychometric network.

Figure 3 illustrates the bootstrapped confidence intervals for edge weights in the psychometric network, reflecting an assessment of the stability and reliability of the connections between variables. The x-axis represents the edge weights, while the y-axis lists the specific node pairs. The black points and lines indicate the bootstrapped mean estimates, while the red points represent the sample estimates. The gray shading around the estimates reflects the confidence intervals derived from the bootstrapping procedure.

#### Bootstrapped confidence intervals

Edges with narrower confidence intervals (closely clustered estimates) indicate higher stability and reliability in a network structure. In contrast, wider confidence intervals suggest greater variability and potential uncertainty in those specific relationships. The strongest and most stable connections appeared on the upper end of the graph (e.g., between self-confidence and efficacy, as well as between the perception, understanding, and expression of one’s emotions and the understanding and expression of others’ emotions), which showed higher positive edge weights with minimal variability. Conversely, weaker or potentially unstable connections appeared lower in the graph, such as between cognitive and affective, which had a negative or near-zero edge weight with wider confidence intervals. Overall, this figure confirmed the robustness of key relationships in the psychometric network while highlighting areas of potential variability that will require careful interpretation in subsequent analyses.

#### Central stability

**FIGURE 4:**
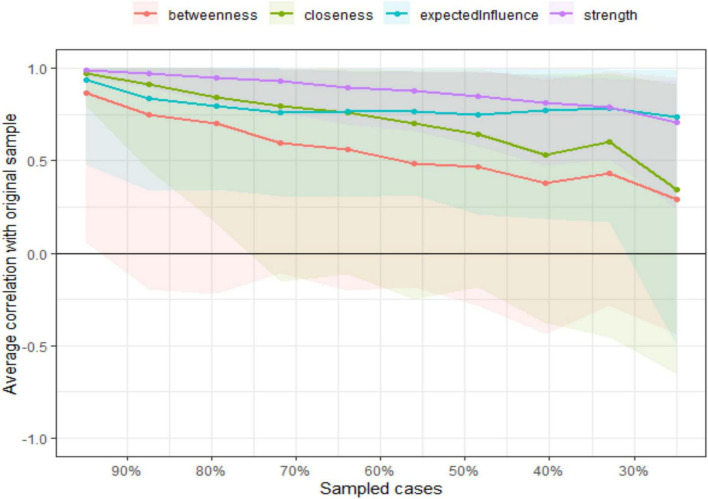
Central stability of network metrics.

Figure 4 illustrates the central stability of the network metrics by showing how the correlation between node centrality indices (betweenness, closeness, expected influence, and strength) and the original sample changed as the number of sampled cases decreased. The x-axis represents the proportion of sampled cases, while the y-axis shows the average correlation with the original sample.

Among the centrality measures, strength (purple) exhibited the highest stability, maintaining a strong correlation even with smaller sample sizes, indicating its robustness in the network. Expected influence (blue) also remained relatively stable, though with slightly more variation at lower sample sizes. Closeness (green) showed moderate stability but began to decline more noticeably as the sample size decreased. Betweenness (red) demonstrated the lowest stability, dropping significantly as the sample size decreased, suggesting that it was more sensitive to sampling fluctuations. This figure confirms that strength and expected influence were the most reliable centrality measures, while betweenness was the least stable, requiring caution in its interpretation in small samples.

## Discussion

This study employed psychometric network analysis to investigate the structural relationships among parental wisdom, psychological resilience, and parent-perceived children’s emotional competence within Saudi Arabian family contexts. Network methodology enabled identification of (a) which constructs function as central hubs exerting disproportionate influence across the psychological system, (b) which specific edges serve as critical bridges connecting parental resources with child developmental outcomes, and (c) the stability of these network structures under varied resampling conditions. The following discussion interprets findings in relation to existing theoretical frameworks, critically evaluates convergences and divergences with prior research, contextualizes results within Saudi family dynamics, and delineates implications for family-centered interventions aligned with Vision 2030 objectives.

### Network structure: three-community architecture and functional clustering

The GLASSO (EBIC) network estimation identified a psychometric structure comprising seven nodes interconnected through 14 edges, yielding an edge density of 0.667. This moderate-to-high density indicates substantial interconnectivity among the measured constructs: parental wisdom dimensions (cognitive, reflective, affective), psychological resilience components (self-confidence, self-efficacy), and parent-perceived children’s emotional competence facets (perception/understanding/expression of one’s emotions [PU_Ones]; understanding/expression of others’ emotions [PU_Others]). Edge weights ranged from −0.104 to 0.598 (mean = 0.156), reflecting heterogeneous association strengths that distinguish central, high-impact connections from weaker, peripheral associations.

Community detection using the Walktrap algorithm revealed three functionally distinct yet interconnected clusters: (1) wisdom (cognitive, reflective, affective components); (2) psychological resilience (self-confidence, self-efficacy); and (3) emotional competence (PU_Ones, PU_Others). This tripartite structure substantiates theoretical propositions that wisdom, resilience, and emotional competence, while related, constitute separable psychological constructs operating through distinct mechanisms (Ardelt, 2003; Luthar et al., 2000; Saarni, 1999). The Louvain algorithm convergently confirmed this multidimensional architecture, and the Total Entropy Fit Index (TEFI = −2.724) indicated that the estimated network represents the observed covariance structure.

The network also exhibited small-world properties [Small-World Index (SWI) = 1.028], characterized by high local clustering and short global path lengths. This topology enhances network efficiency, enabling rapid propagation of influence across nodes. For example, activation of a highly central node such as self-efficacy may quickly impact peripheral constructs, including cognitive wisdom, via short paths. This property provides a data-driven rationale for targeting central nodes in interventions, maximizing network-wide benefits with minimal direct manipulation of all constructs.

Compared with previous studies that examined pairwise relationships (wisdom–resilience: Peixoto et al., 2023; resilience–emotional competence: Schneider et al., 2023), the present network analysis simultaneously models all three domains, revealing how they organize into a coherent yet differentiated system. The observed clustering challenges models that collapse these constructs into a single “positive psychological functioning” dimension. Instead, the network demonstrates that wisdom operates primarily through cognitive-reflective processes, resilience functions via efficacy-based adaptive capacities, and emotional competence reflects parent-perceived child socioemotional abilities. This distinction highlights the importance of tailoring assessment and intervention strategies to each domain, even while recognizing their functional interconnections through network edges.

### Key findings: central nodes as network hubs

Network centrality analyses revealed that self-confidence and self-efficacy exhibited the highest structural importance across multiple metrics, establishing them as critical hubs within the family psychological network. Specifically:

#### Strength centrality

Self-confidence (1.008) and self-efficacy (0.952) demonstrated the highest values, indicating that they maintained the strongest aggregate connections to other network nodes. Their cumulative edge weights were nearly twice the network average, underscoring their role as densely interconnected hubs.

#### Expected influence centrality

Self-efficacy (0.818), PU_Ones (0.797), and affective wisdom (0.781) showed the highest positive expected influence, signifying that activation of these nodes would produce the strongest propagating effects throughout the network. Notably, self-efficacy’s expected influence slightly exceeded its strength centrality (0.818 vs. 0.952), suggesting its edges carry particularly strong positive weights rather than merely numerous connections.

#### Betweenness centrality

Self-confidence (7) and self-efficacy (6) exhibited the highest betweenness, indicating they most frequently lie on shortest paths between other nodes, thereby functioning as critical bridges connecting distinct psychological domains. Affective wisdom demonstrated moderate betweenness (4), suggesting a secondary bridging role, while cognitive wisdom, reflective wisdom, and PU_Others exhibited zero betweenness, positioning them as peripheral nodes dependent on intermediaries for network-wide influence.

#### Closeness centrality

Self-confidence (0.022) and self-efficacy (0.022) displayed the highest closeness, reflecting their efficiency in reaching other nodes via short paths. This metric reinforces their hub status—changes in these constructs can rapidly propagate to all other network elements with minimal intermediary steps.

These converging centrality metrics empirically establish self-confidence and self-efficacy as the network’s core hubs, exerting disproportionate influence on overall system functioning. This extends Bandura’s (1997) social cognitive theory, demonstrating that self-efficacy functions as a network-level lynchpin rather than only an individual-level predictor. In practical terms, enhancing parental self-efficacy and confidence may produce cascading effects across interconnected constructs, including wisdom dimensions, resilience, and perceptions of children’s emotional competence.

An important observation concerns the peripheral positioning of cognitive and reflective wisdom components. Despite theoretical emphasis on these dimensions (Ardelt, 2003; Sternberg, 1998), they showed the lowest betweenness (0) and below-average strength centrality (cognitive: 0.656; reflective: 0.510). Within this network, abstract knowledge, problem-solving abilities, and reflective perspective-taking appear to exert limited direct influence on other psychological domains or child outcomes unless mediated through affective wisdom or efficacy-based action.

These findings highlight the functional importance of affective and efficacy-related constructs within Saudi family systems, while suggesting that cognitive and reflective wisdom—though theoretically valuable—may not directly drive network-wide processes. Further research using culturally grounded measures of wisdom could clarify whether this pattern reflects cultural conceptualizations of wisdom or measurement limitations in Western-derived scales.

### Critical bridge connections: pathways linking psychological domains

Edge betweenness centrality analysis identified the most influential connections facilitating information transfer between network communities. The self-confidence–self-efficacy edge demonstrated the highest betweenness (10) and the strongest edge weight (0.60), marking it as the primary bridge linking resilience components.. This suggests that parents’ global self-confidence and domain-specific parenting efficacy are tightly coupled, mutually reinforcing constructs rather than independent resources.

The bidirectional nature of the edge reflects symmetric association rather than causal direction; increases in one construct are likely to correlate with increases in the other. Practically, interventions that enhance either construct—through skill-building workshops, mentoring, or cognitive-behavioral strategies—may produce collateral improvements in the other, explaining why diverse parenting programs often achieve similar outcomes (Barlow et al., 2012; Chen et al., 2001).

The second-strongest bridge connected self-efficacy and PU_Ones (betweenness = 8), linking the resilience and emotional competence communities. This edge indicates that parents’ confidence in their parenting abilities is directly associated with perceptions of children’s abilities to perceive, understand, and express their own emotions. While the cross-sectional design prevents establishing causal direction, the bridge underscores functional importance: enhancing parental efficacy may positively influence parent-perceived child emotional competence, either by improving emotion socialization practices or by increasing parents’ observational confidence.

The affective wisdom–self-confidence bridge (betweenness = 6) connected the wisdom and resilience communities. This edge reflects that parents’ empathic attunement, compassion, and emotional warmth (affective wisdom) are associated with greater self-confidence. This pattern aligns with attachment-based parenting frameworks, suggesting that parents who experience themselves as emotionally attuned may derive confidence from these qualities, while confident parents may more effectively express affective warmth.

By contrast, cognitive and reflective wisdom components exhibited minimal bridge function (betweenness = 1), indicating that analytical reasoning and perspective-taking contribute little to connecting network communities. This finding challenges the assumption that wisdom’s cognitive dimensions are integrative within family psychological systems. Instead, the results suggest that affective wisdom serves as the primary wisdom-based bridge, consistent with emerging evidence that emotional facets of wisdom are functionally more significant than cognitive facets in close relationship contexts (Ardelt and Jeste, 2018).

These findings highlight key leverage points for interventions: strengthening self-efficacy and affective wisdom may enhance network-wide outcomes, improving parental resilience and perceptions of children’s emotional competence, whereas interventions targeting cognitive wisdom alone may have limited systemic influence without integration with affective or efficacy-related processes.

### Path length analysis: proximal and distal construct relationships

Shortest path analysis provided complementary insights into network topology by quantifying how efficiently activation spreads between nodes. The self-confidence–self-efficacy dyad exhibited the shortest path length (1.63), confirming their tight coupling as immediately adjacent constructs requiring minimal intermediation. Similarly, the PU_Ones–PU_Others connection exhibited a short path length (2.12), indicating that parents’ perceptions of children’s self-focused emotional competencies are directly linked to perceptions of other-focused competencies. This finding aligns with emotional competence theories, which conceptualize these facets as interrelated rather than independent domains (Saarni, 1999).

In stark contrast, cognitive wisdom and PU_Others exhibited the longest path length (15.83), followed by reflective wisdom and PU_Ones (18.58). These extended paths indicate that parents’ cognitive knowledge, analytical abilities, and reflective perspective-taking remain highly distal from parent-perceived child emotional competencies, requiring numerous intermediary nodes for any influence to manifest. Substantively, this suggests that parental intellectual wisdom does not directly shape perceptions of children’s emotional abilities; instead, any cognitive wisdom influence must flow through affective wisdom, efficacy, or confidence intermediaries—a multi-step process susceptible to attenuation.

This pattern has direct intervention implications: programs solely enhancing cognitive wisdom (e.g., teaching problem-solving strategies, providing child development knowledge) may produce minimal child emotional competence benefits unless such cognitive enhancements translate into affective attunement and efficacy-based action. The long cognitive-to-emotional path distances suggest that traditional didactic parenting education—focused on transmitting knowledge—requires supplementation with experiential components cultivating affective wisdom and efficacy to impact child outcomes meaningfully.

### Wisdom-resilience-emotional competence synergies: community structure and clustering

Despite the network’s three-community architecture, within- and between-community edge patterns revealed substantial cross-cluster connectivity, indicating that wisdom, resilience, and emotional competence do not operate in isolation. The moderate edge density (0.667) and small-world properties (SWI = 1.028) demonstrate a balance between functional clustering and efficient inter-cluster communication, enabling systemic coordination across constructs.

The resilience community (self-confidence and self-efficacy) emerged as the integrative core, exhibiting the highest betweenness and connecting both wisdom and emotional competence clusters. This positioning supports resilience theories suggesting that adaptive capacities mediate the effects of distal resources (e.g., wisdom) on proximal outcomes (e.g., child competence) (Luthar et al., 2000). Influence from wisdom components flows primarily through resilience nodes, establishing resilience as a necessary intermediary rather than merely a parallel resource.

Within the wisdom cluster, affective wisdom directly connected to resilience (affective–self-confidence edge), whereas cognitive and reflective wisdom exhibited weaker, indirect links. This heterogeneity suggests that interventions fostering affective wisdom (empathy, compassion, emotional attunement) may be more effective in enhancing parental resilience and, subsequently, parent-perceived child emotional competence than interventions targeting cognitive wisdom alone.

While previous studies (e.g., Golestanibakht et al., 2022) conceptualized emotional competence primarily as an outcome of parental resources, the undirected nature of network edges indicates potential bidirectional influence. The moderate-to-strong connections between PU_Ones and both self-efficacy and affective wisdom suggest that perceiving children as emotionally competent may reinforce parental efficacy and compassion, consistent with transactional family models (Sameroff, 2009). However, this interpretation remains tentative without longitudinal or experimental data; future cross-lagged network studies are needed to clarify the direction of effects.

### Network stability: robustness and limitations of centrality estimates

Bootstrapped case-dropping analyses evaluated the reliability of network centrality metrics. The correlation stability (CS) coefficient indicated that dropping up to 5.2% of the sample maintained centrality rank-order correlations above 0.70 in 95% of bootstrapped samples. This falls well below the recommended threshold of 0.50 (Epskamp et al., 2018), signaling that centrality estimates—particularly betweenness—are sensitive to sample fluctuations.

Strength centrality remained highly stable, preserving strong correlations with original estimates even with reduced sample sizes, confirming it as the most robust metric. Expected influence demonstrated moderate-to-good stability, with minor variability in smaller subsamples. Closeness centrality decreased in stability as sample size declined. Betweenness centrality showed the lowest stability, dropping substantially with fewer participants.

Identification of self-confidence and self-efficacy as central hubs relies primarily on strength and expected influence, which were acceptably stable, supporting confidence in these hub interpretations. Betweenness-based claims regarding bridge edges (e.g., self-confidence–self-efficacy) require caution; replication with larger samples is necessary for definitive conclusions. Despite low CS for betweenness, the overall network backbone—three-community structure, small-world topology, and strongest edges—remained stable. Bootstrapped confidence intervals for edge weights confirmed that the strongest edges (self-confidence–self-efficacy; PU_Ones–PU_Others) were robust, while weaker edges (e.g., cognitive–affective) were less stable. This indicates that core network interpretations are reliable, but peripheral edges should be interpreted carefully.

Comparatively, the observed CS coefficient is lower than typical clinical psychology network studies (median CS ≈ 0.50–0.70; Beard et al., 2016). Network stability is known to depend on sample size, network density, and edge weight distribution (Epskamp et al., 2018). Given the present moderate edge density (0.667) and heterogeneous weights (−0.104 to 0.598), achieving CS > 0.50 would likely require *N* > 400–500 participants. Future research should prioritize larger Saudi samples to strengthen stability and refine centrality-based conclusions.

### Methodological considerations and construct validity concerns

Beyond stability limitations, several methodological considerations temper interpretation:

First, the reliance on parental self-report for all constructs introduces shared method variance potentially inflating edge weights. Parents completing wisdom, resilience, and child emotional competence scales sequentially may exhibit response consistency biases, implicit theories linking constructs (“Wise parents should have emotionally competent children”), or mood-congruent reporting (positive affect elevating all ratings), artificially strengthening observed associations. While GLASSO regularization partially addresses spurious edges by shrinking weak associations to zero, shared method variance affects systematically rather than randomly, potentially surviving regularization. Incorporating multi-informant data (child self-reports of emotional competence, partner reports of parental wisdom/resilience) or behavioral observations would mitigate this limitation and test whether network structures remain stable across informants.

Second, the use of the URPEKD—a scale explicitly designed for parents to assess children’s emotional competence (Mikèević-Milković et al., 2020)—as the sole child competence measure constitutes a construct validity concern. Parental perceptions may not accurately reflect children’s actual emotional abilities due to limited observational access (parents miss school/peer contexts), projection (attributing own emotional states to children), or social desirability (overstating child competencies). While parental perceptions hold pragmatic importance (parents act on their perceptions when making child-rearing decisions), the network edges linking parental wisdom/resilience to parent-perceived child competence may partly reflect cognitive biases rather than substantive parent-child influence processes. For instance, the affective wisdom–PU_Ones association could emerge because compassionate parents genuinely foster child emotional skills *or* because compassionate parents systematically perceive children more positively regardless of actual abilities.

Disentangling perception from reality requires incorporating child self-report emotional competence measures, teacher reports, or behavioral assessments (e.g., emotion recognition tasks). If network structures differ across informants—for example, if parental wisdom associates strongly with parent-reported but not child-reported emotional competence—this would suggest that observed edges partly capture parental cognitive schemas rather than objective child development. Conversely, convergent multi-informant networks would strengthen claims that parental resources genuinely influence child socioemotional capacities.

Third, cultural measurement equivalence remains uncertain. Although instruments demonstrated adequate internal consistency in Arabic translation (McDonald’s ω = 0.728–0.924), factor structures and item meanings may not fully align with Saudi cultural constructs. For instance, wisdom conceptualizations in Islamic intellectual traditions emphasize * _._hikmah* (divinely inspired wisdom, prophetic guidance) and *’aql* (balanced reason-emotion integration) alongside cognitive-reflective-affective dimensions measured by Western 3D-WS scales (Ardelt, 2003), potentially creating construct underrepresentation. If the 3D-WS omits culturally central wisdom facets valued in Saudi contexts, observed cognitive/reflective wisdom peripherality may reflect measurement inadequacy rather than substantive unimportance. Future research employing indigenous Saudi Arabian wisdom measures—developed through emic cultural frameworks incorporating Islamic scholarship on * _._hikmah*—would strengthen construct validity and cultural appropriateness, potentially revealing different network structures when culturally aligned assessments are used.

### Cultural contextualization: Saudi family dynamics and network interpretation

The present study’s Saudi Arabian context introduces cultural considerations potentially shaping how wisdom, resilience, and emotional competence manifest and interconnect within family systems. Saudi families are experiencing rapid societal transitions—economic diversification under Vision 2030, educational reforms emphasizing socioemotional learning, evolving gender role expectations with increasing female workforce participation, and shifts from extended to nuclear family structures (Alenezi et al., 2024). These transformations generate novel parenting stressors: navigating child-rearing in urban nuclear households without traditional extended family support, preparing children for uncertain economic futures diverging from parents’ experiences, and balancing cultural-religious values with globalizing influences.

Within this transitional context, certain network features assume culturally specific significance. The centrality of self-efficacy and self-confidence may reflect adaptive responses to changing parenting demands. As traditional child-rearing models rooted in intergenerational transmission and unquestioned authority become less viable, parents increasingly rely on efficacy beliefs—confidence in their capacity to navigate unfamiliar parenting challenges—as psychological anchors. Efficacy-building interventions might thus address transition-specific anxieties (“Can I prepare my children for careers that don’t exist yet?” “How do I balance religious values with modern educational content?”) particularly salient in rapidly transforming societies experiencing cultural-economic disequilibrium.

The strong affective wisdom bridges to resilience and emotional competence may reflect culturally valued parenting qualities emphasized in Islamic frameworks. Islamic parenting scholarship highlights *ra _._hmah* (compassion), *shafqah* (tender mercy), *rifq* (gentleness), and * _._hilm* (forbearance) as core parental virtues modeled after prophetic teachings (Alqahtani, 2020; Badri et al., 2020). These qualities map conceptually onto affective wisdom dimensions (compassionate concern, empathic understanding), potentially explaining why affective components demonstrated stronger centrality and bridging functions than cognitive components in this Saudi sample. Parents striving to embody Islamic parenting ideals may prioritize affective attunement and compassionate engagement, translating these values into enhanced perceptions of children’s emotional competence through emotionally responsive caregiving and validation.

Conversely, the peripheral positioning of cognitive and reflective wisdom might reflect cultural cognitive styles or measurement issues. Some cross-cultural research suggests that collectivist, relational cultures emphasize contextual, practical, and interpersonally embedded wisdom over abstract analytical reasoning privileged in Western individualist contexts (Takahashi and Overton, 2005). If Saudi parents conceptualize wisdom primarily as relational sensitivity, ethical conduct, and situation-appropriate action rather than logical problem-solving or metacognitive reflection, Western cognitive/reflective wisdom scales may inadequately capture valued capacities. Alternatively, Islamic epistemologies integrating reason (*’aql*) with revelation (*wa _._hy*) and transmitted knowledge (*naql*) might produce wisdom conceptualizations diverging from secular Western models emphasizing autonomous critical thinking.

However, these cultural interpretations remain speculative without comparative data. To substantiate cultural specificity claims, future research should: (a) replicate network analyses in diverse cultural contexts (other Arab nations, Muslim-majority societies, Western populations) to test whether self-efficacy centrality and cognitive wisdom peripherality generalize or represent Saudi-specific patterns; (b) conduct cognitive interviews with Saudi parents regarding wisdom constructs to identify culturally meaningful dimensions potentially absent from Western scales; and (c) develop culturally grounded measures incorporating Islamic psychological concepts (* _._hikmah*, *taqwā*, *tawakkul*) to test whether alternative networks emerge when culturally congruent assessments replace Western-derived instruments.

### Implications for family-centered interventions and vision 2030 alignment

Network analysis findings offer actionable guidance for designing evidence-based family interventions optimized through centrality-based targeting and bridge-focused mechanisms. Three intervention strategies emerge as priorities aligned with Vision 2030s family strengthening and human development objectives:

First: Self-efficacy and self-confidence enhancement as leverage point interventions.

Given these constructs’ demonstrated centrality (strength: 1.008 and 0.952; betweenness: 7 and 6) and their function as the network’s primary hub connecting all communities, interventions strengthening parental efficacy beliefs and confidence may yield disproportionate benefits propagating throughout the psychological system via the network’s small-world topology (SWI = 1.028). Mastery experiences (structured parenting skill-building with successful application opportunities), vicarious learning (peer parent modeling), verbal persuasion (professional affirmation of competencies), and physiological/affective state management (stress reduction to prevent efficacy-undermining anxiety) constitute evidence-based efficacy-building techniques readily adaptable to Saudi contexts (Bandura, 1997).

Practically, community center-based parenting programs could employ Saudi parent facilitators demonstrating effective emotion coaching, conflict resolution, and positive discipline strategies while participants practice with personalized feedback (mastery experiences). Video-recorded testimonials from Saudi parents successfully navigating parenting challenges could provide vicarious learning aligned with cultural communication styles. Islamic family counselors could frame efficacy development within religious frameworks emphasizing *tawakkul* (trust in divine support while taking action) and parental *amānah* (trust/responsibility for children)—reframing efficacy not as secular self-reliance but as confidence in fulfilling divinely ordained parental roles with divine assistance. Such culturally adapted efficacy interventions could leverage religious motivation and community support systems inherent in Saudi society.

Network centrality suggests such programs may produce cascading improvements extending beyond direct skill acquisition: enhanced efficacy should strengthen wisdom application (particularly affective wisdom given the self-confidence–affective bridge), reinforce resilience through positive adaptation feedback, and improve perceived child emotional competence via the efficacy–PU_Ones bridge—all through network propagation even without explicitly targeting these constructs. This network-informed approach maximizes intervention efficiency by concentrating resources on high-leverage central nodes rather than diffusing efforts across all possible parenting domains.

Second: Affective wisdom cultivation to strengthen parent-child emotional transmission pathways. Affective wisdom’s bridging function (betweenness = 4) and moderate-to-high expected influence (0.781) position it as a secondary intervention target linking the wisdom community to resilience and emotional competence outcomes. Interventions incorporating mindfulness-based compassion training, emotion coaching skill development, and reflective parenting approaches could enhance parental empathy, emotional attunement, and compassionate responding—affective wisdom’s core components.

Culturally adapted implementations might draw from Islamic contemplative practices: *murāqabah* (mindful awareness of divine presence), *tadabbur* (reflective Quranic meditation), and *tafakkur* (contemplation of creation) could serve as culturally congruent mindfulness analogues fostering present-moment awareness and compassionate perspective-taking (Badri et al., 2020). Parenting workshops teaching *ra _._hmah*-based responding to child distress, implementing the prophetic model of gentle child interaction (*rifq*), and developing * _._hilm* (patient forbearance) with children’s developmental limitations would resonate with religious values while cultivating affective wisdom. Such culturally embedded framing may enhance engagement and reduce perceptions of Western secular impositions potentially resisted in conservative Saudi contexts.

Given affective wisdom’s bridge to perceived child emotional competence, programs cultivating parental compassion and empathic attunement should theoretically enhance parents’ recognition and fostering of children’s emotional capacities—either through genuinely improved emotion socialization practices or through compassion-enhanced perceptual sensitivity to children’s emotional expressions, or both. The network structure suggests this pathway operates independently of cognitive parenting knowledge, reinforcing that emotionally attuned presence may benefit children as much or more than didactic expertise.

Third: Integrated resilience-wisdom programs recognizing synergistic network clustering. Although community detection differentiated wisdom and resilience constructs, their substantial intercommunity connectivity—particularly affective wisdom’s resilience bridges—suggests integrated programs simultaneously cultivating both domains may prove more effective than single-construct interventions through mutually reinforcing mechanisms. For example, resilience training teaching cognitive reappraisal (“Viewing setbacks as growth opportunities”) naturally cultivates reflective wisdom’s perspective-taking, while wisdom practices examining life’s complexity and uncertainty enhance resilience through meaning-making and acceptance. Vision 2030 family support initiatives could prioritize such integrated programs over fragmented workshops addressing isolated skills.

Practically, an integrated curriculum might sequence wisdom and resilience modules synergistically: initial sessions establishing self-efficacy foundations (foundational given centrality), followed by reflective wisdom practices enhancing perspective-taking during conflicts (supporting cognitive reappraisal resilience), then affective wisdom cultivation through compassion training (reinforcing emotional resilience and bridging to child outcomes). This sequencing aligns with network topology—building the central hub first, then extending to connected nodes through established pathways, maximizing cumulative benefit propagation.

These intervention strategies directly operationalize Vision 2030s Quality of Life Program objectives targeting resilient families, empowered individuals, and societal wellbeing. By concentrating on network-identified leverage points (central nodes, bridge edges) rather than diffusing resources across all conceivable parenting domains, implementation can achieve population-level impact with constrained budgets—a critical consideration for scaling family support services nationwide. Network-informed resource allocation enables evidence-based prioritization: invest in self-efficacy programs first given centrality, supplement with affective wisdom training to activate bridges, integrate resilience components recognizing clustering synergies.

## Limitations and future research directions

Several limitations temper interpretation and generalizability of findings.

First, the cross-sectional design precludes causal inference regarding edge directionality. While network edges represent partial correlations controlling for all other variables—thereby reducing spurious associations from unmodeled confounds—they remain symmetric and cannot establish whether, for example, self-efficacy enhances affective wisdom, affective wisdom boosts efficacy, or bidirectional influences operate. Temporal network analysis employing intensive longitudinal designs (daily diaries, ecological momentary assessment) can map within-person dynamics over time, revealing temporal precedence and potential causal chains (Bringmann et al., 2013). Such designs could test whether efficacy boosts precede next-day wisdom application, wisdom practice enhances subsequent efficacy, or synchronous reciprocal effects occur—critical for intervention timing decisions.

Second, shared method variance from exclusive parental self-report potentially inflates edge weights and creates spurious network connections. Multi-informant network models comparing parent-report, child-report, and observer-based structures can identify measurement-specific artifacts versus substantive relationships. If self-efficacy centrality emerges only in parent-report networks but not in multi-informant networks, this would suggest reporter bias rather than genuine hub status. Conversely, convergent structures across informants would substantiate validity.

Third, the URPEKD’s parental proxy measurement of children’s emotional competence introduces potential perception-reality discrepancies. Parents high in wisdom/resilience may systematically overestimate child abilities through positive schemas, while distressed parents may underestimate competencies through negative biases, creating edges reflecting cognitive processes rather than child development. Parallel assessment using child-report emotional competence scales (appropriate for children 10+ years, comprising 57.5% of the present sample) and behavioral tasks (emotion recognition accuracy, emotion regulation strategy effectiveness) would enable perception-reality comparison and test whether parental resource–child outcome edges differ across informants.

Fourth, sample characteristics limit generalizability. The predominantly 40–49-year-old, 11–20-year-married parent sample represents mid-life family stages; networks may differ for younger parents navigating early intensive child-rearing (higher stress, lower wisdom accumulation) or older parents with emerging adult children (different parent-child dynamics). Recruitment via digital and community channels may have selected more educated, urban, psychologically minded parents, underrepresenting rural, low-literacy, economically marginalized, or culturally conservative populations. Multi-site replication across diverse Saudi socioeconomic strata, geographic regions (rural vs. urban), and family structures (single-parent, blended, multigenerational households) is essential for population-representativeness claims and equitable intervention design avoiding middle-class urban bias.

Fifth, the low centrality stability coefficient (CS = 5.2%, far below the 0.50 threshold) necessitates cautious interpretation of centrality rankings, particularly betweenness-based bridge identifications. While strength and expected influence demonstrated relative robustness, betweenness proved highly unstable. Replication with *N* > 400–500 Saudi parents should prioritize achieving CS > 0.50 before making definitive centrality-based intervention recommendations. Additionally, cross-validation approaches (split-sample network estimation, out-of-sample edge weight prediction) could assess whether the network generalizes beyond the estimation sample.

Sixth, cultural measurement equivalence remains uncertain. Western-derived scales, despite Arabic translation and adequate reliability, may not capture culturally central constructs or may measure culturally peripheral aspects. Indigenous Saudi Arabian instrument development incorporating Islamic psychological concepts (* _._hikmah*, * _._habr*, *taqwā* for wisdom; *tawakkul*, *ri _._hā* for resilience; *adab*, *i _._hsān* for emotional competence) could reveal alternative network structures when culturally congruent assessments replace Western imports. Qualitative research exploring Saudi parents’ emic conceptualizations of wisdom, resilience, and emotional competence should precede scale development to ensure cultural validity.

Seventh, network estimation method choices (GLASSO with EBIC tuning vs. alternative regularization; Gaussian assumption for ordinal Likert data) involve analytical decisions potentially influencing results. Sensitivity analyses examining whether network structures remain stable across estimation methods (GLASSO vs. nonparanormal transformations, EBIC vs. cross-validation tuning), edge weight computation approaches (partial correlations vs. regularized regression), and centrality indices (expected influence vs. strength vs. hybrid metrics) would strengthen confidence that findings reflect genuine psychological structures rather than methodological artifacts.

Finally, the present network was estimated at the scale level rather than the item level, which may introduce the “fallacy of the averages” (Epskamp et al., 2018). While this approach enables a parsimonious examination of inter-dimensional dynamics and conceptual relationships, it may obscure item-level bridges or finer-grained associations. Future research with larger samples could implement item-level network analyses to capture these subtler patterns and validate the robustness of the current scale-level findings.

Future research priorities include: (1) Temporal network analysis through intensive longitudinal designs tracking how parental resources and child competence co-evolve, enabling within-person causal process identification. (2) Multi-informant network models incorporating child self-reports, partner reports, teacher reports, and behavioral observations to validate parent-report structures and identify informant-specific biases. (3) Intervention studies experimentally manipulating central nodes (efficacy enhancement programs) or bridge edges (affective wisdom training) to test whether predicted network propagation effects (cascading benefits to non-targeted constructs) materialize, providing causal validation of centrality-based targeting. (4) Cross-cultural network comparisons across Arab nations (Gulf Cooperation Council countries, Levant, North Africa), Muslim-majority societies (Malaysia, Indonesia, Turkey), and Western contexts (US, Europe) to identify universal vs. culturally specific family psychological dynamics. (5) Network modeling integrating additional ecological variables (marital quality, socioeconomic stress, extended family support, community cohesion, religious involvement) to situate wisdom-resilience-emotional competence networks within broader family system contexts. **(6)** Indigenous measurement development employing mixed methods (qualitative interviews, cognitive testing, psychometric validation) to create culturally grounded Saudi Arabian family psychology assessments capturing constructs potentially absent from Western scales.

## Conclusion

This study advances family psychology and intervention science by providing the first psychometric network analysis of parental wisdom, psychological resilience, and parent-perceived children’s emotional competence within Saudi Arabian family contexts. Key empirical contributions include: (a) identification of self-confidence and self-efficacy as central network hubs (strength centrality: 1.008 and 0.952; betweenness: 7 and 6) suggesting high-leverage intervention targets; (b) documentation of the self-confidence–self-efficacy edge as the network’s strongest bridge (edge weight = 0.60, betweenness = 10) connecting resilience to other psychological domains; (c) revelation of affective wisdom’s critical bridging function (betweenness = 4) linking wisdom to resilience and child emotional competence while cognitive/reflective wisdom remained peripheral; (d) demonstration of small-world network topology (SWI = 1.028) enabling efficient information propagation; and (e) three-community clustering (wisdom, resilience, emotional competence) indicating functional differentiation despite substantial intercommunity connectivity.

Theoretically, findings challenge simplistic unidirectional models assuming parental characteristics straightforwardly determine child outcomes, instead revealing complex bidirectional networks where parent and child constructs mutually influence one another through reciprocal feedback loops and bridge pathways. The observed network structure suggests that parental psychological resources do not operate as independent predictors but as dynamically interconnected system elements whose collective configuration determines family functioning—a systems perspective with direct intervention implications.

Methodologically, the study demonstrates network analysis’s value for identifying intervention leverage points through empirically derived centrality metrics rather than a priori theoretical assumptions. However, the low centrality stability coefficient (CS = 5.2%) underscores the necessity of larger samples (*N* > 500) for definitive centrality ranking in future Saudi family research, while convergent findings across strength and expected influence metrics lend confidence to core hub identifications despite overall stability limitations.

Culturally, results highlight potential divergences from Western family psychology: self-efficacy/confidence centrality and cognitive wisdom peripherality may reflect Saudi collectivist parenting contexts emphasizing role fulfillment and affective attunement over individualist analytical reasoning. Alternatively, measurement non-equivalence from Western-derived scales could produce these patterns, necessitating indigenous instrument development before drawing firm cultural conclusions.

Practically, findings offer evidence-based guidance for Saudi family policy aligned with Vision 2030s family strengthening objectives: (1) prioritize self-efficacy enhancement as foundational interventions given centrality and cascading network potential; (2) cultivate affective wisdom through culturally adapted compassion training (*ra _._hmah*-based approaches) to strengthen parent-child emotional transmission bridges; (3) implement integrated wisdom-resilience programs recognizing synergistic clustering rather than fragmented single-skill workshops; and (4) frame interventions within Islamic parenting frameworks emphasizing divine trust (*tawakkul*), parental responsibility (*amānah*), and prophetic compassion models (*ra _._hmah*, *rifq*) to enhance cultural resonance and community engagement.

Within the Vision 2030 framework prioritizing human capital development, family resilience, and quality of life enhancement, these network-informed intervention strategies enable resource optimization: by concentrating efforts on empirically identified leverage points (central nodes, critical bridges) rather than diffusing investments across all parenting domains, family support programs can maximize population-level impact with constrained budgets—critical for achieving nationwide scale and sustainability. Future research replicating these networks in larger, more diverse Saudi samples; extending analyses through longitudinal designs and experimental interventions; incorporating multi-informant and culturally grounded assessments; and conducting cross-cultural comparisons will further refine understanding of family psychological networks and enable increasingly precise, culturally responsive, evidence-based interventions supporting Saudi families navigating rapid societal transformation while preserving cultural-religious values and fostering children’s optimal socioemotional development.

## Data Availability

The raw data supporting the conclusions of this article will be made available by the authors, without undue reservation.
